# Unambiguous observation of blocked states reveals altered, blocker-induced, cardiac ryanodine receptor gating

**DOI:** 10.1038/srep34452

**Published:** 2016-10-05

**Authors:** Saptarshi Mukherjee, N. Lowri Thomas, Alan J. Williams

**Affiliations:** 1 Wales Heart Research Institute, Cardiff University School of Medicine, Heath Park, Cardiff, CF14 4XN, United Kingdom

## Abstract

The flow of ions through membrane channels is precisely regulated by gates. The architecture and function of these elements have been studied extensively, shedding light on the mechanisms underlying gating. Recent investigations have focused on ion occupancy of the channel’s selectivity filter and its ability to alter gating, with most studies involving prokaryotic K^+^ channels. Some studies used large quaternary ammonium blocker molecules to examine the effects of altered ionic flux on gating. However, the absence of blocking events that are visibly distinct from closing events in K^+^ channels makes unambiguous interpretation of data from single channel recordings difficult. In this study, the large K^+^ conductance of the RyR2 channel permits direct observation of blocking events as distinct subconductance states and for the first time demonstrates the differential effects of blocker molecules on channel gating. This experimental platform provides valuable insights into mechanisms of blocker-induced modulation of ion channel gating.

Ion channel gates act as dynamic barriers to modulate the flow of permeant ions through the channel pore, thereby fulfilling critical physiological roles in cellular signal transduction. Extensive structure-function studies in the last two decades, especially on prokaryotic K^+^ channels, have resulted in the identification of the determinants of gating^1^,^2^,^3^. The inner helix bundle crossover region (IHBx) and the selectivity filter (SF) in the channel pore are currently thought to be the primary components of the gating mechanism^4^ and these could also be functionally coupled in some ion channels^5^.

While ionic flux through the pore is controlled by the gates, the ion occupancy itself was found to have an effect on channel gating behaviour by bringing about subtle conformational changes in the SF region, a phenomenon that has been the focus of several recent studies^6^,^7^,^8^. The role of ion occupancy in SF-mediated gating has been investigated by altering the concentration of ions as well as by replacing the permeant ion (usually K^+^) with other monovalent and divalent cations to bring about changes in pore conformation and affect gating^9^,^10^. Large tetra-alkyl ammonium cations (TAAs), which are pore blockers in K^+^ channels, have also been employed as probes to study the effects of resulting perturbations in ion occupancy on channel gating^11^,^12^,^13^,^14^. This potentially useful technique is, however, not without caveats. When bound TAAs, fully occlude K^+^ channel pores, leaving no residual current, and so making the differentiation of blocking and true closing events difficult in single channel current data. In studies involving some K^+^ channels, longer closing events in single channel recordings are assumed to occur due to pore block (effect on ion permeation) by TAAs^14^,^15^ but there is also a possibility of them arising as a result of a direct effect of the blocking molecule on channel gating that stabilises closed conformations. As a result, the assignment of state transitions in gating models emanating from such experiments is not straightforward.

The cardiac ryanodine receptor (RyR2) is a massive (~2.2 MDa) Ca^2+^ release channel responsible for transducing the information from an incoming action potential to trigger cardiac contraction^16^. It is a Ca^2+^-activated cation selective channel that exhibits a large single channel conductance for K^+^ (~723 pS in 210 mM K^+^) and the pore is only partially occluded by blocking TAAs, resulting in distinct subconductance states due to the presence of residual currents^17^,^18^,^19^. In the absence of structural details from x-ray crystallographic data of the pore forming region (PFR) of RyR2, recent functional studies point to the possibility of there being two gates in the channel pore^20^. The Ca^2+^ (ligand) controlled gate formed by the IHBx was found to be mechanistically distinct from gating at the putative SF region which is ligand independent. Using the detailed tertiary structure of the bacterial K^+^ channel KcsA^2^ as a template, a highly plausible model of the putative RyR2 PFR was constructed using the last two transmembrane helices of RyR2 along with the luminal loop^21^. This showed that the overall structural arrangement of the key elements of the RyR2 pore analogy model closely resembles the known structure of KcsA and could contribute to channel function in a similar manner^22^. Recent high-resolution electron cryomicroscopy (cryo-EM) data from RyR1 channels also supports this putative configuration of the channel pore^23^.

The ability to clearly distinguish between blocked and closed events due to differences in current amplitudes (subconductance states) in RyR2 presents a massive advantage over the situation in K^+^ channels and prompted this study in which the role of the SF along with the mechanisms of blocker-induced effects on channel gating (direct and/or electrostatic) could be examined. This experimental platform uses RyR2 and the TAA blockers tetrabutyl- (TBA) and tetrapentyl ammonium (TPeA) to study block at the single channel level, aided by hidden Markov model (HMM)-based analysis programs for accurate detection and modelling of state transitions. This study demonstrates that block of RyR2 by TAAs modify the channel’s gating behaviour and reveals differences in the mechanisms of blocker interaction within the pore and their differential effects on channel gating.

## Methods

### Expression and purification of recombinant RyR2

The protocols for expression of recombinant mouse RyR2 in HEK293 cells and its subsequent purification for single channel experiments in planar phospholipid bilayers have been described in detail in a previous study^19^. Briefly, HEK293 cells were transfected by mouse wild-type (WT) RyR2 cDNA using Ca^2+^ phosphate precipitation and cells were harvested after 48 hours of growth. The transfected cells were homogenised and solubilised using 0.6% (w/v) 3-[(3-cholamidopropyl) dimethylammonio]-1-propanesulfonic acid (CHAPS) and 0.3% L-α-phosphatidylcholine (PC) at a protein concentration of 2 mg/ml. The solubilised supernatant obtained after removal of insoluble material using low-speed centrifugation (14000 g), was carefully layered onto a continuous sucrose density gradient (5–40%) and spun at 100,000 g for 17 hours at 4 °C. Sucrose fractions containing channel proteins were identified by incorporation into lipid bilayers, before being snap frozen in small aliquots in liquid nitrogen and stored at −80 °C until use.

### Single-channel recording

Planar phospholipid bilayers were formed from suspensions of phosphatidylethanolamine (Avanti Polar Lipids, AL, USA) in *n*-decane (35 mg/ml) across a 200 μm diameter hole in a polystyrene copolymer partition that separates two solution-filled chambers, *cis* (volume 0.5 ml) and *trans* (volume 1 ml). The *trans* chamber was held at virtual ground while the *cis* chamber could be clamped at various holding potentials relative to ground. Current flow across the bilayer was measured using an operational amplifier as a current-voltage converter^24^,^25^. Bilayers were formed in solutions containing 600 mM KCl, 20 mM HEPES, titrated to pH 7.4 with KOH, resulting in a recording solution containing 610 mM K^+^ in both chambers (symmetrical solutions). Single channels were incorporated into the bilayer by adding aliquots of RyR2-containing fractions to the *cis* chamber after establishing a *cis*-*trans* osmotic gradient by adding 1–2 aliquots (50–100 μl) of 3 M KCl to the *cis* chamber^25^. Under these conditions, channels usually incorporated into the bilayer within 1–3 minutes upon stirring the *cis* chamber, after which further incorporations were prevented by removing excess unincorporated channel proteins and symmetric ionic conditions were reinstated by perfusion of the chamber with 610 mM K^+^ solution. RyR2 channels incorporate into the bilayer in a fixed orientation so that the cytosolic face of the channel is exposed to the solution in the *cis* chamber and the luminal face to the *trans* chamber^26^. All experiments were carried out at room temperature (21 ± 2 °C).

The effects of large TAA blockers TBA and TPeA (Sigma-Aldrich) were examined after their addition to the solution in the *cis* chamber, as these molecules are known to block from the cytosolic side of RyR2 when the direction of ionic flux is from the cytosolic to the luminal side^18^,^19^,^27^,^28^. The probability of detection and quantification of blocked events was optimised by using experimental conditions that favour channel opening to enable blocker entry into RyR2 pore. This was achieved by activating the channel using 150 μM EMD41000 (an isomazole analogue), added to the *cis* chamber^25^,^29^. The high permeant ion concentration (610 K^+^) that improves the signal-to-noise ratio, along with the increased channel open probability (Po) of EMD41000 (EMD) activated RyR2 enables accurate resolution of blocking events in single channel current traces. Only recordings where single RyR2 channels had incorporated into the bilayer were analysed and traces containing multiple channels were discarded.

### Data acquisition and analysis

Single-channel current fluctuations were low-pass filtered at 5 kHz with an 8-pole Bessel filter and then digitised at 20 kHz with a PCI-6036E AD board (National Instruments, Austin, TX). Acquire 5.0.1 (Bruxton, Seattle, WA) was used for viewing and acquisition of the single-channel current fluctuations. Data analysis was carried out using the QuB suite of programs version 2.0.0.14 (http://www.qub.buffalo.edu) and has been described in detail in our previous studies^20^,^30^.

Briefly, single-channel current traces of 1–2 minutes were idealised, employing the segmental K-means algorithm based on Hidden Markov Models. A dead time of 120 μs was imposed during idealisation of the single channel current recordings that resulted in the calculation of the mean amplitude, the Po, median open (T_o_) and closed (T_c_) durations, along with frequency of closings. The open and closed dwell-time histograms generated by the initial idealisation were fitted with a mixture of exponential probability density functions using the maximum interval likelihood function of QuB. The maximum interval likelihood (MIL) program simultaneously optimises the rate constants for the transitions between the states during fitting of dwell-time histograms by computing the maximum log likelihood (LL) of the idealised data, given a model. The program also corrects for missed events that are shorter than the imposed dead time. A minimum of 8000 events were utilised from each experiment for estimation of single channel kinetic parameters and at least 2000 events used for the computationally intensive fitting and modelling tasks. Results are expressed as mean values ± S.E.M and a t-test was applied for analysing statistical significance.

## Results

### Activation of RyR2 for visualisation of block

Single channel Po must be sufficiently high for accurate detection and quantification of block from the fully open state. Single mouse recombinant RyR2 channels incorporated in planar lipid bilayers under symmetric ionic conditions (610 mM KCl; cytosolic/luminal) were therefore first activated using EMD (cytosolic) before TAA blockers could be added to the cytosolic side. Previous studies have established that TAAs are able to block RyR from the cytosolic side, only when the direction of ionic flux through the channel pore is from the cytosolic to the luminal side^18^,^19^,^27^,^28^. In this study, we have examined the effects of blocker interaction with the pore on RyR2 gating behaviour for each EMD-activated single channel at positive holding potentials (cytosolic-luminal current) when compared with data acquired at corresponding negative holding potentials (luminal-cytosolic current) when there is no block. The latter served as an effective internal control in the experiments and the EMD-activated single channel gating kinetics in the absence of block will serve as a platform upon which the effects of blocker on single channel gating behaviour of activated RyR2 could be unravelled.

**Figure 1:**
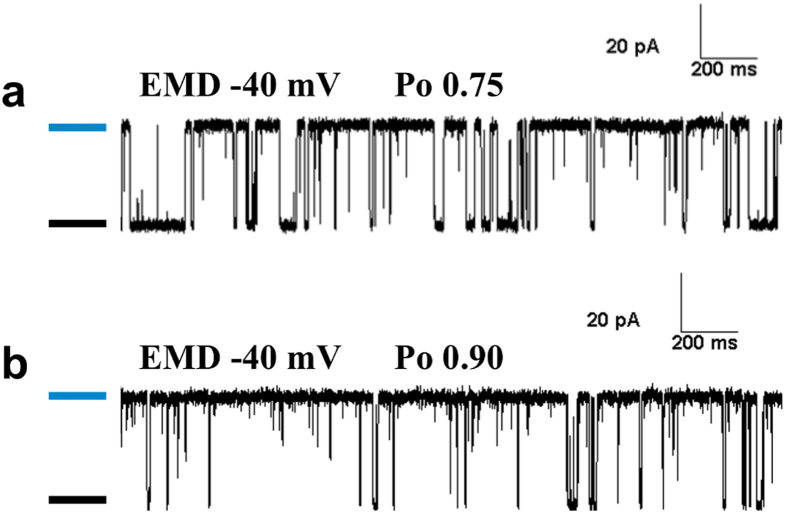
Activation of single RyR2 channels by EMD. (**a**,**b**) are two representative single-channel current traces where single RyR2 channels incorporated in planar lipid bilayers were activated using EMD (cytosolic) in the absence of block. The membrane potential was clamped at −40 mV where ionic flux was in the luminal (*trans*) to cytosolic (*cis*) direction with opening events as upward deflections from the closed level (marked by black bars) to the fully open level (blue bars). The channels can be activated by EMD to varying degrees as shown by their respective open probabilities (Po).

**Figure 2:**
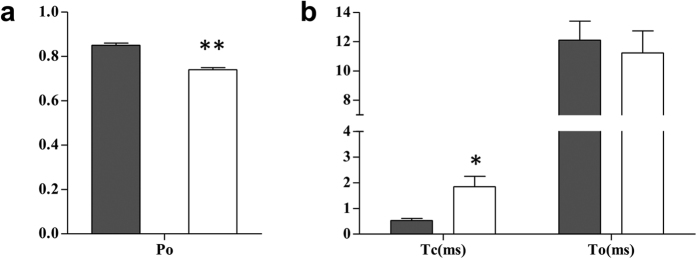
Comparison of single channel kinetic parameters of RyR2 activation. Single channel parameters were grouped according to their Po as shown in (**a**), where dark bar represents group of channels with Po ≥ 0.8 and white bar represents relatively lower activity channels (0.7 ≤ Po ≤ 0.79). The variations in Po (**p = 0.001; n = 5) are mainly as a result of alterations in channel closed times (Tc) (*p < 0.01; n = 5), while open times (To) remain unchanged as shown in (**b**).

EMD-activated channels having high Po (Po ≥ 0.7) were used for modeling single channel gating behaviour to study the effect of blocker molecules as sufficient blocked events can be accurately observed under such conditions. Representative channel data traces from EMD-activated single channels at −40 mV holding potential (in the absence of block) are shown in Fig. 1. Single channel kinetic parameters from these channels were grouped according to their activities (Po 0.7–0.79 and ≥ 0.8, Fig. 2a) to demonstrate that variations in Po are primarily due to changes in closed times (Tc), while open times (To) remained unaffected (Fig. 2b).

### Modelling the gating behaviour of EMD-activated RyR2

**Figure 3:**
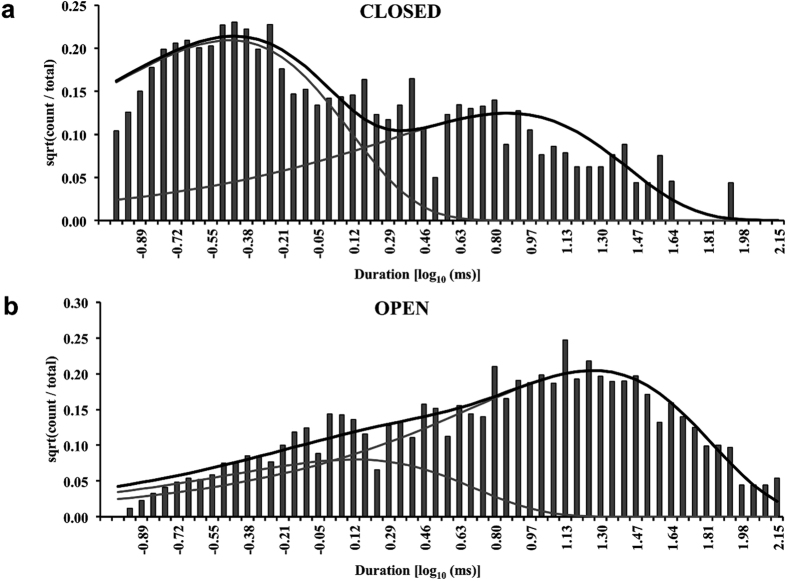
Dwell-time histograms and their respective exponential fits. Representative closed and open time distributions are shown in (**a**) and (**b**) respectively, where the grey lines represent individual exponential fits of the histograms and black lines show the overall summation of fits. Both open and closed time histograms are each shown to have two distinct distributions for EMD-activated RyR2 channels, suggesting a four-state gating scheme.

**Figure 4:**
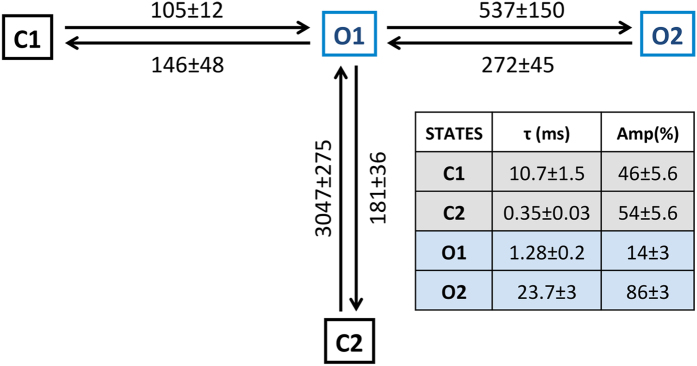
Gating scheme describes EMD-mediated activation of RyR2 channels. Two open and closed states each were required as minimum for a putative gating model that is shown along with the rate constants for state transitions (data derived from 8 different single channel experiments). The parameters of exponential fits from these channels (time constant **τ** and fractional area (**Amp**)) are shown in the inset table. Data represented as mean values ± S.E.M (n = 8).

The events detected by idealisation of single channel data (Po ≥ 0.7) were used to generate closed and open dwell-time histograms that were then fitted with exponential probability density functions (see Methods). Representative closed and open time histograms constructed for EMD-activated RyR2 channels along with their exponential fits are shown in Fig. 3, which indicate that a minimum of two closed and open states each are required to accurately describe single channel kinetics under these conditions. The closed-time distributions (Fig. 3a) have a slightly higher proportion (54 ± 5.6%; n = 8) of brief closing events (τ = 0.35 ± 0.03 ms) when compared to longer closed events (τ = 10.7 ± 1.5 ms; n = 8) while events with longer open times (τ = 23.7 ± 3 ms; n = 8) dominate (86 ± 3%) the open-time distributions (Fig. 3b). The MIL program of QuB software allows for the simultaneous optimization of transition rate constants between various states in the model derived from fitting of the dwell-time histograms (see Methods). Supplementary Table 1 shows the top four gating schemes ranked according to their maximum LL values that are generated after fitting dwell-time distributions from single channel data. EMD-activated channels have a simplified putative gating model (Fig. 4) with only two closed (C1 & C2) and two open states (O1 & O2) as opposed to channels activated by Ca^2+^ alone, where a minimum of 3 closed and 4 open states are required^20^ for an accurate description of gating behaviour. The most likely kinetic scheme (top-ranked model in Supplementary Table 1) derived from 8 EMD-activated RyR2s at −40 mV along with the rate constants of transitions are shown in Fig. 4 as well as the parameters obtained from exponential fits of the dwell-time histograms (inset table). In the gating model (Fig. 4), the longer closed state C1 directly communicates with the short-lived open state O1 (τ = 0.35 ± 0.03 ms; n = 8) which is then able to change its conformation to a much longer duration open state O2 (~24 ms). The channel can make brief sojourns from O1 to a second closed state C2 that is short-lived (~0.35 ms) but direct transitions between the two closed states (C1 and C2) are not favoured under these experimental conditions. A previous study has suggested that these brief flicker-closing events from the open state to a short-lived closed state (C2) are the manifestation of channel gating at the selectivity filter (SF) region^20^. The equivalent ligand/voltage-independent transitions in K^+^ channels are thought to be due to the meta-stable nature of the SF and related to the ion occupancy of this flexible region^31^. In this study, the ligand EMD is used purely as a RyR2 activator in order to achieve a range of single channel open probabilities with an objective of measuring effects of QA blockers on channel gating. Therefore, the gating models and their respective state transition topologies are phenomenological descriptions of channel behaviour during activation and will assist in the elucidation of possible modulation of such activity during block.

### RyR2 gating and blocker access to the channel pore

Organic cations like large TAAs can only gain access to the RyR2 ion conduction pathway with the opening of the gate formed by the putative inner helix-bundle crossover (IHBx). This mechanism of pore-entry and block had been predicted by earlier studies on K^+^ channels^1^,^12^ and has been shown in crystal structures of ion channel pores with blockers *in situ*^32^,^33^,^34^. However due to the lack of direct information from crystal structures regarding the determinants of gating in RyR, it is envisaged that the study of blocker kinetics may shed further light on the molecular mechanism of gating in the channel. Some recent studies examining the RyR using single-particle cryo-EM support the existence of an IHBx in the ion permeation pathway that may be involved in gating^23^,^35^.

**Figure 5:**
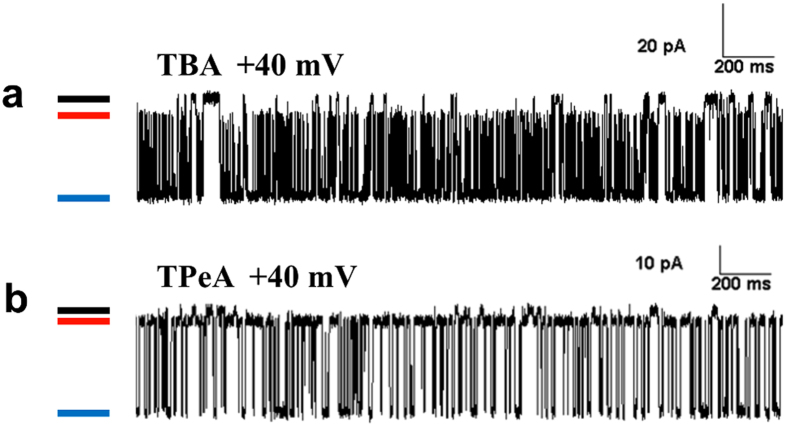
RyR2 single channel currents blocked by TAAs allow residual ion permeation. (**a**,**b**) are representative single channel traces showing EMD-activated single RyR2 channels that are blocked to subconductance states using cytosolic TBA (200 μM) and TPeA (100 μM) respectively at a holding potential of +40 mV. The black bars mark the baseline (closed level), while channel openings are downward deflections to the fully open level (shown by blue bars). The red bars show the subconductance levels that are as a result of pore block by the TAA molecules.

**Figure 6:**
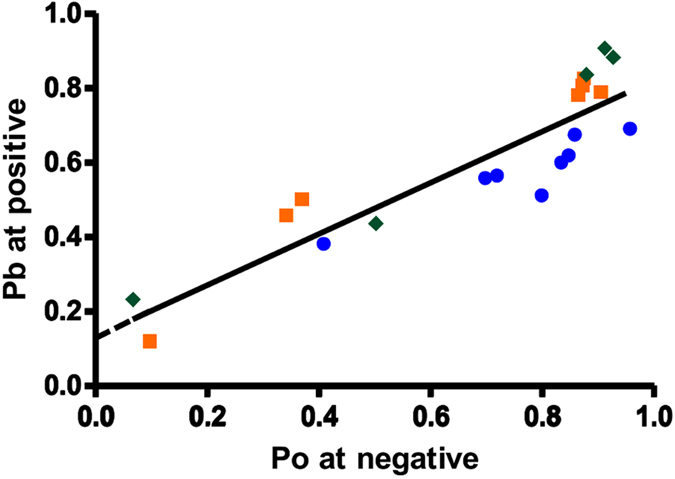
TPeA blocker activity and its relationship with channel Po at various holding potentials. The Pb due to TPeA at various positive holding potentials was plotted against the Po of the same channels (EMD-activated) at corresponding negative holding potentials (data derived from 5–8 single channel experiments for each holding potential). The linear regression through the points shows that the access to the pore is provided by the opening of the ligand-dependent gate at the IHBx. The y-axis intercept of the extrapolated line (dotted) indicates the presence of another gate which could also contribute to channel activity. Symbols representing the data points are as follows: 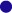 ± 40 mV, 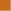 ± 50 mV and 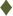 ± 60 mV.

The representative traces in Fig. 5a,b depict EMD-activated single RyR2 channels that are subsequently blocked to subconductance states using cytosolic TBA (200 μM) and TPeA (100 μM) respectively at a holding potential of +40 mV. The fractional conductances of the blocked states induced by the molecules have been well characterised in previous studies^18^,^19^,^27^,^28^ at ~20% of control for TBA and ~14% for the larger TPeA. These studies have also examined the voltage dependence of block of various TAA compounds where it was found that the K_off_ decreased with increasing holding potentials while the effect on K_on_ was not so pronounced. Transitions between the blocked and the closed states are well resolved in the single channel data and will enable us to describe channel gating with the blocker molecule *in situ*. The action of the blockers on RyR2 channels activated to varying degrees by EMD was studied at different positive holding potentials to quantify the propensity of block with respect to its ease of access to the pore. Single channel Po observed at corresponding negative holding potentials (in the absence of block) quantifies the access provided to the blocker molecule by opening of the gate formed by the putative IHBx. The probability of block (Pb) obtained at positive holding potentials (+40 to +60 mV) plotted against single channel Po at corresponding negative holding potentials is shown for the blocker TPeA (Fig. 6). EMD-activated RyR2 channel gating is not voltage dependent *per se* as has been shown in a previous study^25^, thus any effect on channel gating observed during block (at positive holding potentials) is due to the blocker itself. The data suggest that the propensity of block is proportional to the access provided to the blocker by opening of the ligand-activated gate, leading to an open channel block. However, extrapolating the line of best fit to the y-axis (dotted line; Fig. 6) indicates that a closed conformation of the channel can be blocked. As the opening of the putative gate at the IHBx (stimulated by the ligand EMD) is essential for the blocker to get into the RyR2 pore, there must be another functionally distinct gate that can restrict the permeation of ions to close the channel and allow blocker access to the closed channel at the same time. Our previous functional studies had suggested the possibility of gating at the SF region of the RyR pore^20^ and the current study provides further evidence in favour of the existence of a distinct ligand-independent gating entity which resides distal to a putative site of blocker action. This phenomenon was also observed in experiments employing the blocker TBA but the channel activities obtained (Po at negative holding potentials) were not variable enough for a suitable graphical representation (see Supplementary Fig. 1).

### Blocker-induced modulation of RyR2 channel gating

**Figure 7:**
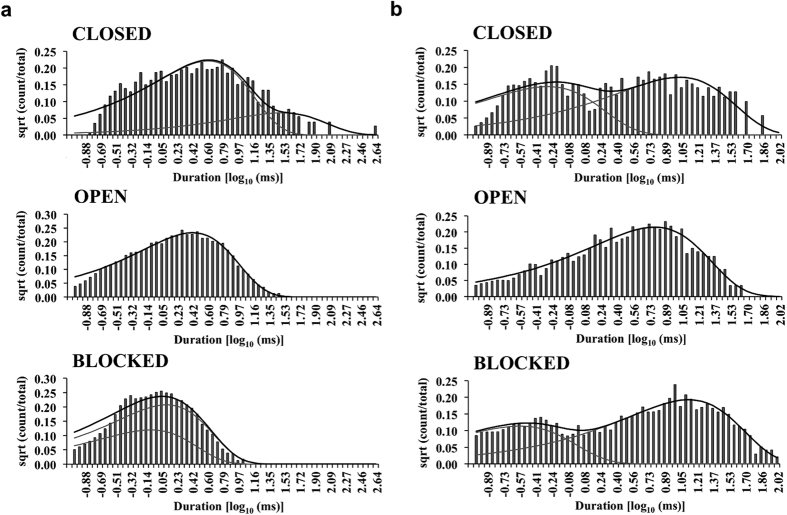
Comparison of dwell-time distributions between the blockers TBA and TPeA. (**a**,**b**) are representative closed, open and blocked dwell time histograms that were derived from single channel experiments using the TAA blockers TBA and TPeA respectively. The grey lines represent the individual exponential fits and the black lines show the overall fits of the distributions. For both TBA and TPeA, two closed, one open and two blocked components were required for the fits suggesting a five-state gating scheme for blocker kinetics. On visual inspection, the closed and the blocked time histograms for the two blockers show major differences in their distribution and individual components of fit. The parameters of exponential fits of the histograms derived from single channel data are shown in Fig. 8 below.

**Figure 8:**
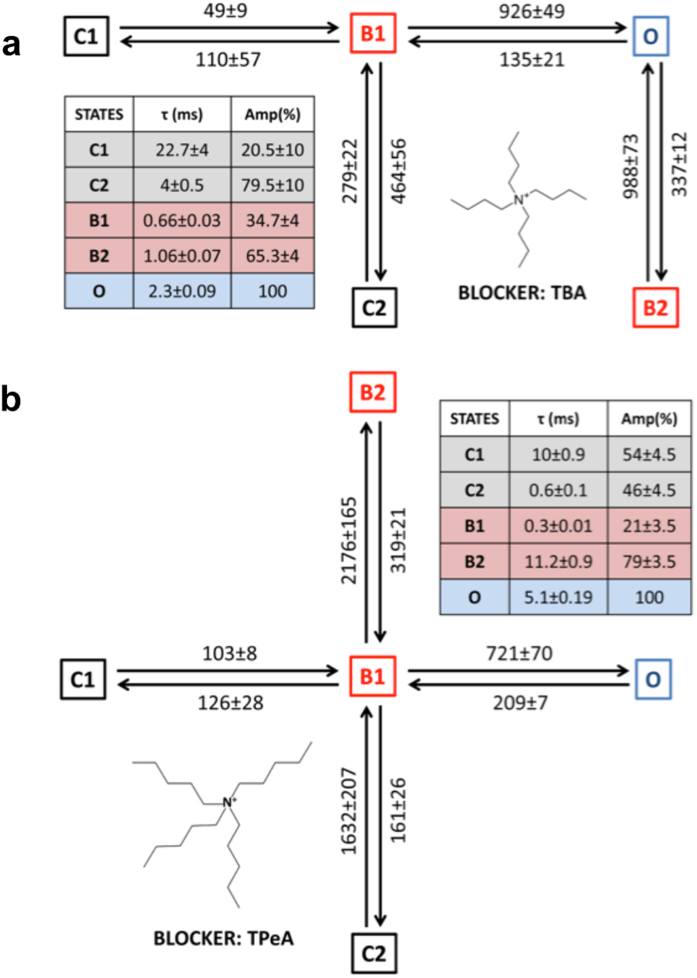
Description of RyR2 gating behaviour in the presence of TAA blockers. (**a**,**b**) shows the gating schemes (top-ranked models in Supplementary Table 1) derived from single channel data for the blockers TBA and TPeA respectively. The rate constants for the state transitions are shown along with the parameters of individual exponential fits (time constants and areas) of dwell-time histograms (inset tables). Data represented as mean value ± S.E.M (TBA, n = 4 and TPeA, n = 5). With either blocker, only one open state is predominantly resolved and no direct transitions are detected between open and closed states. The blocked states (B1 & B2) can both communicate through the open state (O) in the case of TBA (**a**), while with TPeA the blocked states can directly communicate with each other (**b**).

Single channel data from this study point towards the existence of two distinct gates in the RyR pore and how channel gating could have a bearing on the mechanism of blocker action. However, a complete understanding of any possible effect of ongoing block on RyR2 gating mechanism requires detailed kinetic modeling of single channel blocker activity. An exercise of this kind is significantly more complex in channels in which blocking molecules fully occlude the pore (e.g. various K^+^ channels) but is feasible in RyR2 because of the very high single channel conductance and the occurrence of subconductance state block with the large TAAs. The blocked states in RyR2 are thus essentially ‘open-blocked’ conformations where the channel gates are still open allowing residual ionic flux though the pore. Idealisation of single channel data using HMM based algorithms allowed us to accurately resolve events due to sojourns between the blocked and the closed states even when the residual current was small (see Supplementary Fig. 2). The dwell time histograms generated from experiments with TBA and TPeA at +40 mV along with the exponential fits are shown in Fig. 7. The parameters of fit and the most likely gating schemes (see Supplementary Table 1 for top four schemes) using simultaneous optimisation of rate constants between closed, blocked and open states are shown in Fig. 8. The open time histograms obtained from data using both blockers could be fitted using a single probability distribution function (Fig. 7) and the gating models incorporate one open state (O) that communicates directly with the blocked states but never with the closed states (Fig. 8a,b). The high concentration of blockers used in single channel experiments for observing frequent detectable blocking events will have resulted in the very low likelihood of resolving direct closed-open transitions in the gating schemes as the probability of a blocker being present in the pore is high.

The most important effect of the block is seen on the closed states in the case of TBA, where the short-lived flicker closing events (τ ~0.35 ms) observed in the absence of block (Fig. 4) are replaced by significantly longer duration closing events (τ ~ 4 ms) as shown in Fig. 8a. However, brief flicker closing events are still observed when TPeA is used as the blocker (Fig. 8b), although they are slightly prolonged (τ ~0.6 ms). Also, the blocked states (B1 & B2) can both communicate directly with the open state (O) in the case of TBA (Fig. 8a), while with TPeA the blocked states can directly communicate with each other (**8b**). The longer residence of blocking TPeA in the pore manifests as sojourns to a substantially longer duration blocked state B2 (τ = 11.2 ± 0.9 ms; n = 5) when compared to TBA (τ = 1.06 ± 0.07 ms; n = 4) which has a higher K_off_, reflecting differences in hydrophobicity between these two TAAs^19^.

These findings reveal a direct effect of large TAA block of the RyR2 pore on channel gating and demonstrate different mechanisms of action of the two blockers used in this investigation. The possible mechanistic implications of the distortions brought about by the blockers on channel gating are discussed further in the next section.

## Discussion

High-resolution structural details of the channel pore derived from K^+^ channels have previously been utilised as templates to generate putative models of the RyR ion permeation pathway^21^,^36^,^37^, with recent cryo-EM and single channel data providing further evidence for generic similarities between the two species of channels^23^. However, in spite of these cation permeable channels having similar structural determinants of gating and permeation such as the SF and IHBx regions, there are pronounced differences in their functional characteristics. The RyR single channel conductance is much larger (723 pS for K^+^ at 210 mM KCl) when compared to any other class of ion channel and almost an order of magnitude greater than that of the K^+^ channel KcsA^38^. However, the inability of TAAs to completely occlude the RyR2 pore, leading to a distinct subconductance state, in contrast to K^+^ channels where the closed and blocked single channel current levels are indistinguishable, provides us with a unique experimental approach to examine channel gating during block. X-ray crystallographic data from various K^+^ channels with large QA blockers *in situ* reveals that the binding site for the blocker molecule is located in the large hydrophobic cavity, close to the cytosolic entrance to the SF^33^,^34^,^39^. The presence of the blocker in this region effectively vacates the SF region of all K^+^ ions and completely obliterates ionic flux through the K^+^ channel^32^, leading to a blocked state that cannot be distinguished from the closed state on the basis of conductance. Recent neutron diffraction and solid-state NMR studies of KcsA demonstrate that the binding of the blocker molecule TBA displaces water from the cytosolic cavity and reduces the ionic flux to zero^40^, providing a plausible mechanism of block by TAAs. The presence of a residual ionic flux through the RyR pore in the presence of the blocker molecule suggests that the displacement of water molecules is probably less effective when compared with K^+^ channels.

**Figure 9:**
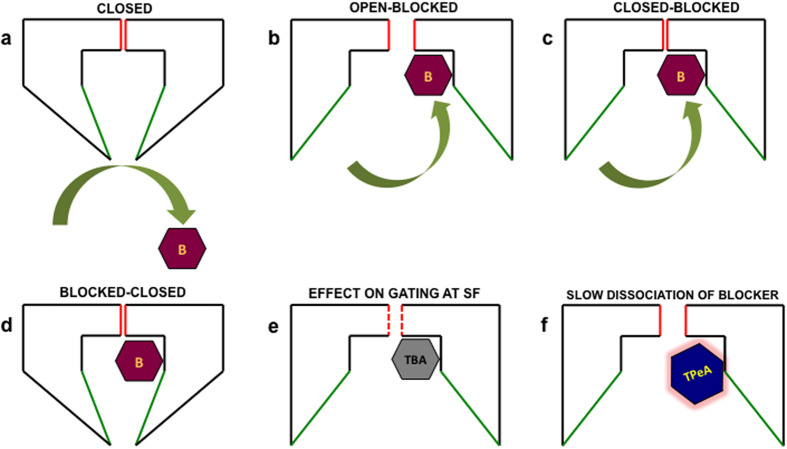
Schematic representations of possible scenarios in TAA blocker interaction with RyR2 pore. The cartoons (**a,b**) show that the access of the generic TAA blocker (B) to the RyR2 channel pore is controlled by the ligand-dependent gate formed by the putative IHBx (shown in green). The blocker can also access a closed conformation of the channel when the gate formed by the SF (shown in red) is closed (no ionic flux) but that formed by the IHBx is open, as shown in (**c**). The blocker-bound channel could also then close (**d**) through the IHBx gate. TBA could be pushed deep inside the channel pore to be able to interact with the SF region (red-dashed) and alter gating (**e**) while the larger TPeA cannot proceed as far as TBA but could reside in the pore for longer (lower K_off_) as in (**f**).

The resolution of distinct closing events from the blocked level in RyR2 single channel data is a significant advantage over other channels, as the various state transitions could then be unambiguously assigned in the kinetic model of block by TAAs. This study shows that increasing the probability of opening of the channel gate (formed by the IHBx)^37^ that is triggered by binding of an activating ligand such as EMD also increases the access of the blocker to the pore, resulting in more frequent blocking events (Fig. 6). However, this result also demonstrates that the blocker could have access to, and block, the channel pore when RyR2 is in a closed confirmation. Although at first this seems to be counterintuitive, the phenomenon could be explained if another gate, that is distal to the blocker binding site in the pore, is considered to be involved in closing the channel while the ligand-activated gate formed by the IHBx remains open, allowing access of the blocker molecule to its binding site (Fig. 9a–c). The SF has been shown to be involved in gating in various K^+^ channels and was also implicated in RyR2 gating in a recent functional investigation^20^. The current study provides further evidence in favour of the existence of a SF gate in the RyR pore, distinct from the gate at the IHBx.

The use of EMD as an activating ligand to increase RyR2 single channel Po for accurate detection of blocking events also simplifies the kinetic schemes that describe channel gating behaviour, requiring only 4 states (Fig. 4) when compared to channels that are solely activated by cytosolic Ca^2+^ where at least 7 states are required^20^. This subsequently provides a robust platform for developing gating models that can describe the effects of TAA blockers on EMD activated RyR2 as it serves to keep the number of required states to a minimum.

The HMM based algorithms used in single channel event detection could accurately assign transitions between the blocked, closed and open states along with their corresponding rate constants (see Methods). The predominant effect of the large QA blocker TBA on RyR2 gating is on the kinetics of channel closings where the typical brief flicker closing events (Fig. 3a) present in the absence of block (state C2 in Fig. 4; τ = 0.35 ± 0.03 ms; n = 8), disappear and are replaced by closings events (C2 in Fig. 8a; τ = 4 ± 0.5 ms; n = 4) of much longer duration (Fig. 7b). As the population of brief flicker closing events has been attributed to ligand-independent channel gating at the SF region in previous functional studies on RyR2^20^, it is likely that TBA interacts with amino acid moieties near its binding site in the cytosolic vestibule below the SF to influence gating at this location, leading to the stabilisation of a closed conformation of longer duration (Fig. 9e). Previous structural studies in K^+^ channels and supporting electrophysiological data suggest possible interaction of TAA blockers with the SF along with permeant ions present in the conduction pathway^13^,^33^. TBA-induced block of KcsA was shown to completely obliterate the brief closing events (τ = 0.5 ms) observed in the absence of blocker. With the blocker present these events are replaced by a much longer non-conducting state^33^ that is not clearly differentiated from the blocked state in KcsA channels as there is no residual current. Similar mechanisms could be envisaged to be playing a role in TBA-induced changes in RyR2 channel gating where blocker molecules are thought to bind to a region consisting of several hydrophobic residues on the inner helix lining the cavity wall^19^,^23^. Interestingly, the larger and more hydrophobic QA blocker molecule TPeA does not significantly distort the population of brief closing events as seen in the dwell time histograms (Fig. 7a) and the resulting gating model (Fig. 8b). Although structure-function and simulation studies in the much smaller K^+^ channels predict that large TAAs share a binding site in the hydrophobic cytosolic vestibule below the SF^33^,^34^,^39^, this may not be the case for a massive channel molecule like RyR where the blocker molecules may bind to different hydrophobic patches lining the putative pore^19^. Therefore it is possible that TBA, being a smaller blocker molecule, can access a binding site that is located deeper in the pore compared to the larger TPeA and, in this location, is able to exert an influence on the SF to affect RyR2 gating. Although some studies in K^+^ channels have shown that the channel gate (IHBx) could close with TAA blocker *in situ* which may perturb gating through a ‘finger in the hinge’ mechanism^34^,^41^, the high Po of EMD activated RyR2 used in the current study prevents the detection of such closed states in the gating models.

On examination of the two blocked states (B1 and B2) resolved in the gating models for each of the blockers TBA and TPeA (Fig. 8), it is evident that they differ in their *modus operandi* in RyR2. The larger and more hydrophobic molecule TPeA can bind with a higher affinity to its non-polar binding site compared to TBA, with higher affinity resulting from a 22-fold difference in the rate of bound blocker dissociation^19^. This manifests as the stabilisation of a much longer blocked state (B2; τ~11 ms) in the TPeA gating model (Fig. 8b) when compared to the corresponding state for the TBA model (B2, τ~1 ms) as shown in Fig. 8a. However, the mechanism of block in RyR2 seems to be more complex as the presence of a residual current allows two distinct blocked states with different dwell-times (B1 and B2) to be resolved in the gating schemes for both blockers, but only in the case of TBA do the block states communicate through the intermediate open state (Fig. 8a). A previous study in KcsA has shown that it is possible for a large QA blocker like TBA to exist in different configurations inside the cytosolic cavity and two such orientations of the molecule were most frequently encountered^33^. This phenomenon could also be applicable in the case of RyR where a larger cytosolic cavity^21^,^37^ could allow different blocker configurations to reside and variably interact with the hydrophobic residues leading to the stabilisation of at least two different blocked conformations of the channel. However, TBA being less hydrophobic, with a higher K_off_, will readily dissociate from its binding site thereby relieving block before binding again in a switched orientation. Such a sequence would produce, the B1 ↔ O ↔ B2 transitions with an intervening open state being resolved (Fig. 8a). The more hydrophobic blocker TPeA interacts with its binding site for a longer period before coming off (low K_off_) which might permit a switch in its molecular configuration *in situ* and stabilisation of a different blocked state without having to pass through an intervening open state (B1 ↔ B2; Fig. 8b).

The novel findings reported here demonstrate that large TAAs not only block RyR2 and modify its gating behaviour, but, in addition, reveal differences in the location of their sites of interaction within the cytosolic vestibule and in their mechanisms of block. The data add weight to our previous conclusion that both the IHBx and SF are determinants of RyR2 gating and accentuate the need for further structure-function studies to unravel the complex interactions between these gates and molecules that influence them.

## Additional Information

**How to cite this article**: Mukherjee, S. *et al*. Unambiguous observation of blocked states reveals altered, blocker-induced, cardiac ryanodine receptor gating. *Sci. Rep.* **6**, 34452; doi: 10.1038/srep34452 (2016).

## Supplementary Material

Supplementary Information
