# Supplementary material for: Unambiguous observation of blocked states reveals altered, blocker-induced, cardiac ryanodine receptor gating
Source: Sci Rep. 2016 Oct 5;6:34452. doi: 10.1038/srep34452 (PMC5050499; doi:10.1038/srep34452)
Supplement: Supplementary Information [file srep34452-s1.pdf]

Supplementary Information

Unambiguous observation of blocked states reveals altered, blocker-induced, cardiac ryanodine receptor gating.

Saptarshi Mukherjee, N. Lowri Thomas and Alan J. Williams

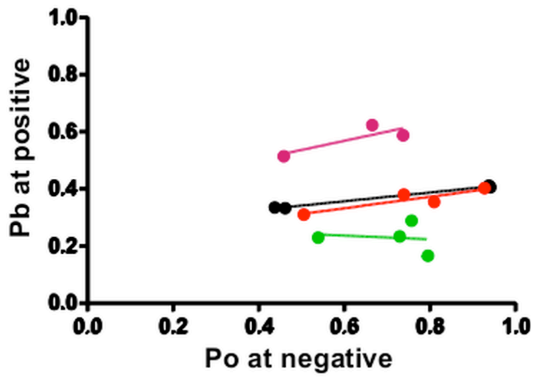

Supplementary Figure 1. Relationship of channel Po with Pb during block by TBA. EMD-activated RyR2 channels were studied at various holding potentials where Po measured in the absence of block (at negative voltages) were plotted against Pb observed at corresponding positive holding potentials when TBA is able to block the channel. The linear relationships between Pb and Po as observed in case of TPeA (see Fig. 6 in Results) are not clear here as the RyR2 activities achieved in these experiments fell within a narrow Po range. 3-4 single channel experiments were conducted for each of the holding potentials where the symbols representing the data points and linear regressions are as follows: ● ±40 mV, ● ±50 mV, ● ±60 mV and ● ±80 mV.

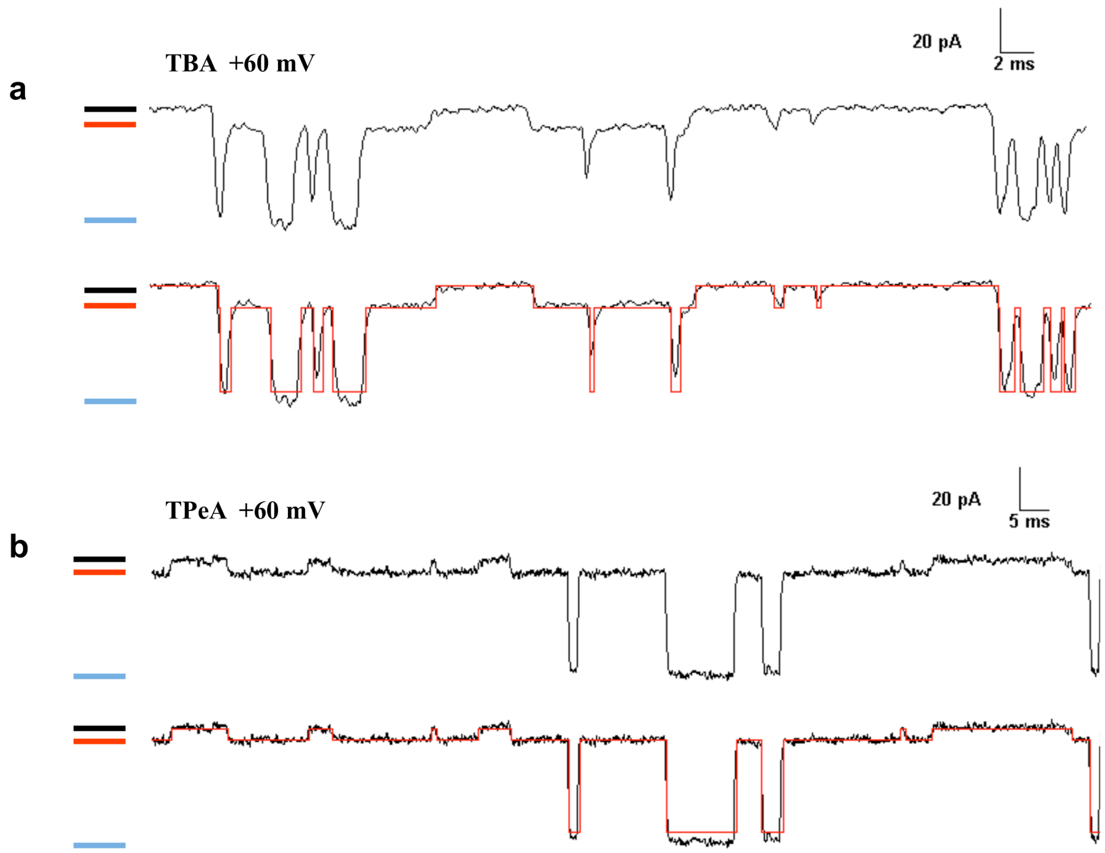

Supplementary Figure 2. Accurate detection of single channel current block events in RyR2 using HMM-based analyses. Single channel current traces at +60 mV are shown with an expanded time base for better visualisation of transitions to and from the blocked states. The closed levels are denoted by the black bars from which channel opening events are downward deflections and the full open channel current levels are marked by the blue bars. The red bars show the subconductance states brought about the blockers occluding the channel pore and are due to the residual currents. The idealisation results in the detection of events as state transitions and is shown by the overlaid red line. (a) and (b) show the raw single current traces and their corresponding idealised traces for the large organic cationic blockers TBA and TPeA respectively.

Gating schemes with maximum LL for ligand and blockers			
Model Rank	EMD41000	TBA	TPeA
I	C1↔O1↔O2<br>↕<br>C2<br>23300	C1↔B1↔O<br>↕ ↕<br>C2 B2<br>71000	B2<br>↕<br>C1↔B1↔O<br>↕<br>C2 8124
II	C1↔C2↔O1<br>↕<br>O2<br>23000	C1↔B1↔O<br>↕ ↕<br>C2 B2<br>70977	C1↔B1↔O<br>↕<br>C2 ↔ B2<br>8104
III	C1↔O1<br>↕<br>C2↔O2 22960	C1↔B1↔O<br>↕<br>C2 ↔ B2 70946	C1↔B1↔O<br>↕<br>C2↔B2 8022
IV	C1↔O1↔O2<br>↕<br>C2 22930	C1↔C2↔B1↔O<br>↕<br>B2 70936	C1↔C2↔B1↔O<br>↕<br>B2 7897

Supplementary Table 1. Gating models derived from fitting single channel data are ranked according to their maximum log-likelihood values. The gating schemes were derived from representative single channel experiments using EMD as the activating ligand (6700 events) and the TAA blockers TBA (13800 events) and TPeA (2110 events). The top-ranked models (highest LL) that are most likely to be accurate descriptions of RyR2 gating behaviour under the specific experimental conditions are described in detail **(Figs. 4** and **8** in Results).
